# Editorial: Lessons and policy consequences of mathematical modeling in relation to ongoing pandemics

**DOI:** 10.3389/fpubh.2023.1281493

**Published:** 2023-10-09

**Authors:** Pierpaolo Ferrante

**Affiliations:** Department of Occupational and Environmental Medicine, Epidemiology and Hygiene, Italian National Workers' Compensation Authority (INAIL), Rome, Italy

**Keywords:** COVID-19 mathematical modeling, COVID-19 policy, global pandemic plan, pandemic containment measures, pandemic preparedness

COVID-19 marked the second pandemic of the 21st century, following the swine pandemic (A/H1N1pdm09) of 2009. It is the third outbreak of coronaviruses after SARS (2003) and MERS (2013), and the seventh monitored outbreak (including those caused by the Zika virus and the avian flus A/H5N1 and A/H7N9). Excluding the Zika virus (belonging to the “Flaviviridae” family) and A/H5N1, the remaining were emerging viruses (1). While some uncertainty persists about the origin of SAR-CoV-2, current evidence suggests an animal origin (2). According to numerous studies highlighting the role of Climate change in increasing the risk rate of cross-species viral transmission (3), the potential for pandemics to emerge as one of the most significant threats to humanity in the future is evident (4). The development of coordinated national pandemic plans should be a priority for every country in order to release a global response to a global issue (5).

An effective pandemic plan should be designed through a multidisciplinary approach, offering flexibility for calibration based on evolving data evidence, and structured around the following points:

(a) Epidemiological: Establish a robust epidemiological surveillance system encompassing the entire national territory. Based on varying assumptions about the virus's virulence, simulated scenarios should be run to project the virus's potential spread within the population. Epidemiological thresholds to activate restrictive public policies (including mobility restrictions and social distancing) as well as mandatory sectorial behaviors (including the use of FFP2 face mask in public transport, smart working, and distance learning) should be proposed based on predictive model outputs. During the pandemic, it is important to adapt the most relevant existing models to data evidence and develop new models as necessary.

(b) Medical: Research and development of vaccines and treatments to address the biological aspects of preventing and caring the disease. Healthcare workers must be trained to manage the pandemic with simulations over time to ensure basic preparedness.

(c) Logistics: Implementing the healthcare response by defining the necessary resources (including diagnostic tests, protective equipment, hospital capacity, and vaccines) and organizing their distribution across territories. Coordination from national government to local institutions with multiple decision-making centers should be implemented to facilitate collective territorial coherence and minimize the possible consequences of government crisis. Primary health care should play an active role in guaranteeing system resilience (6).

(d) Political and ethical: Selecting the epidemiological thresholds and determining their implementation pattern requires a delicate balance among competing human rights, including liberty, economy, and health. Sociologists, jurists, and constitutionalists should engage in relevant discussions aimed at increasing the likelihood of public acceptance.

(e) Communication: Design a communication campaign using multimedia platforms to effectively convey accessible and clear information in a visually and verbally engaging manner, reaching a wide audience. Emphasize scientific dissemination rather than TV entertainment programs and address the anti-vax problem with the appropriate information.

This Research Topic includes 11 original research, two brief reports, two reviews, and one perspective paper. All the original research and brief reports either used publicly available data or were made accessible upon request to the authors. Four studies included the analysis code. Simulations were carried out through the COVASIM model (7), which is implemented in free python code.^1^ Focusing on mathematical modeling, the collected papers addressed points (a) and (e) of the previous list during the COVID-19 pandemic.

The primary overarching conclusion from this Research Topic is the remarkable proliferation of mathematical modeling during the emergency period. This global effort reflects the impressive mobilization of human societies worldwide as well as an underlying lack of preparedness and coordination. For instance, the Israeli health response relied on three different models with varying assumptions and outcomes (Niv-Yagoda et al.).

An overview of pandemic characteristics was provided through descriptive and predictive models. By analyzing cases from the Mexico's surveillance system during the first 2 years, Loza et al. confirmed the key role of comorbidities in disease severity and the effectiveness of vaccination campaigns. Ferrante introduced the negative binomial model to estimate the incidence of infection from mortality in Italy. Results indicate that over 40% of infections went undetected, with the majority occurring before the introduction of rapid tests. Cumsille et al. predicted the occupancy of intensive care units by adding to the SIR model a compartment representing the number of patients in intensive care and two parameters describing the rate from susceptible to recovered (due to vaccine protection) and the vice versa (due to vaccine immunity decay).

A description of the models used to evaluate the effectiveness of the vaccination campaign along with two study using them are included. Filho et al. conducted a review on studies addressing the impact of a vaccination program. They found that half of them simulated scenarios with and without vaccines, while the others compared the populations before and after vaccination. By simulating the scenario without a vaccination, Ferrante found that vaccines prevented 115,000 deaths during the first two pandemic years in Italy. By comparing the pre- and post-vaccination populations, Lokonon et al. studied the lag-time effects of vaccination through a quasi-Poisson regression with a distributed lag linear model. They found a significant protective effect when the 40% of people were vaccinated, with a lag time of 15 days for the effect of the third dose.

Non-pharmaceutical interventions were extensively investigated, including their impact on seasonal influenza. Montcho et al. analyzed these interventions using a distributed lag linear model. They found that stricter restrictions led to fewer admissions in regular and intensive care units, with a 9–10 day time lag. Rodríguez et al. investigated the impact of non-pharmaceutical interventions in Spain using a data-driven agent-based model. Simulations revealed that the combination of tracing and testing, along with the associated isolation of positive individuals, halved infections and deaths. Valgañón et al. investigated the socioeconomic determinants of stay-at-home through a SEIR model that included a permeability parameter and a predeceased compartment. Their study highlighted the need for equitable global policies, showing the challenges low-income countries face in mitigating the virus spread and protecting vulnerable populations. Lin et al. studied the effects of non-pharmaceutical policies on the seasonal flu and found that wearing face masks and avoiding crowded places protected ~20 and 40% of people, respectively. Furthermore, if more than 85% of people had adopted both behaviors the reproduction number could have been <1.

As with other respiratory viruses, schools played a relevant role in the COVID-19 spread. Yin et al. studied the university resumption impact using a disaster management perspective and the pressure–state–response model. Their model included six factors representing disaster hazards that university can only monitor (including epidemic risk level of the school's location and means of transportation back to school) and fourteen factors related to system vulnerability that can also be controlled (including student behaviors and routine campus activities). Through simulations, Abeysuriya et al. compared three testing strategies in schools: home quarantine of all contacts of a positive case; “test-to-stay” strategy for close contacts of a case for 7 days; and an asymptomatic surveillance strategy involving twice-weekly screening of all students. Compared to extended home quarantine, test-to-stay strongly increased days of face-to-face teaching while maintaining a similar effectiveness for reducing school infections. Asymptomatic screening was beneficial in reducing both infections and lost days of face-to-face teaching especially when community prevalence was high.

The COVID-19 pandemic also marked an intensive use of machine learning and sentiment analysis in epidemiological modeling. Varón et al. performed a review to describe the role of machine learning in health policies. They found an increasing usage of these methods both in COVID-19 and long COVID studies, including clinical diagnosis, epidemiological analysis, drug discovery, patterns and relationships of symptoms, and predicting risk indicators. Chen et al. proposed integrating epidemic modeling with content and sentiment infoveillance based on natural language processing. They concluded that infoveillance from massive social media data complements and enhances current epidemic models. Zhang et al. conducted a sentiment analysis of the Chinese reopening policy after 3 years of “zero-COVID” measures. They found a negative attitude toward “sudden” measures and suggest preparing people in advance with relevant health consultation services and an effective communication strategy.

Results from this Research Topic suggest several points to upgrade the pandemic response from national to a global level. In particular, for improving the initial preparedness we recommend:

1) WHO should lead research efforts to identify and classify potential spillover viruses (8) and advance research on related vaccines and therapies.
2) WHO should develop guidelines for establishing public and standardized national-level epidemiological virus surveillance systems based on Statistical Data and Metadata eXchange (9). These systems ensure the collection of consistent national data, which can be simply transmitted to a global database (such as the global influenza surveillance and Response System) and made accessible through user-friendly APIs.
3) National pandemic plans should include simulations of virus spread, considering varying levels of virulence and transmission abilities. These simulations should use an agent-based model and encompass factors such as the saturation level of hospitals and the impact of pharmaceutical and non-pharmaceutical policies.
4) Countries should integrate pandemic-era hygiene rules (such as wearing face mask in crowded places and washing hands after touching surfaces potentially contaminated) into primary school hygiene education.

Additionally, priorities during a pandemic should include:

5) In countries initially affected, lethality should be promptly estimated through community serosurveys focused on the area surrounding the initial deaths. Subsequent refinement can be achieved through regional and national serosurveys that track virus circulation.
6) Estimating the lethality hazard ratios for the virus variants compared to the original strain, along with the relative risks of infection and death among vaccinated and unvaccinated individuals, will allow for the consistent application of the negative binomial model throughout the entire pandemic period.
7) Ensure the rapid availability of reliable rapid tests to support a worldwide testing campaign for identifying positive cases and contact tracing.
8) Secure the swift availability and equitable global distribution of effective vaccines.
9) Calibrate health policy and communication campaigns based on sentiment analysis to increase public compliance.

## Author contributions

PF: Conceptualization, Writing—original draft, Writing—review and editing.

## Footnotes

^1^Available online at: https://github.com/InstituteforDiseaseModeling/covasim.
